# Blunted Loss Reactivity in Subclinical Bipolarity During Risky Choice

**DOI:** 10.1192/j.eurpsy.2026.11202

**Published:** 2026-07-31

**Authors:** I. Kasikci, E. Yilmazbas, Z. Çeliköz

**Affiliations:** 1 Psychology; 2 Neuroscience, Bahsecehir University, Istanbul; 3 Department of Psychology, Bahçeşehir University, İstanbul, Türkiye

## Abstract

**Introduction:**

Risk-taking is shown to be common in bipolar disorder (BD). We hypothesized that the alignment (or misalignment) between expected and immediate affective responses can shape risky choice. We examined the expectation–experience gap in subclinical bipolar disorder (SBD) as a putative mechanism relevant to risky decision-making.

**Objectives:**

To quantify and compare the difference between expected and immediate affect (negative and positive) across monetary loss and win outcomes in SBD versus a control group, and to assess whether group differences emerge—especially for loss-related responses.

**Methods:**

Fifty-five adults (SBD = 27, control group = 28, age range= 18-45) completed a coin-toss gambling task arranged as eight rounds of five tosses each (40 total). Outcomes were pre-generated so that each participant completed two low-loss, two high-loss, two low-win, and two high-win rounds. On each round, participants provided expected affect ratings before the five-toss sequence and immediate affect ratings after the sequence (both negative and positive; 5-point Likert scale). SBD status was defined using Bipolar Prodrome Scale (BPS) cut-offs (Frequency ≥18 or Severity ≥40; Turkish validation by Aydemir et al., 2018). We targeted a subclinical sample to probe early mechanisms and minimize confounds from medication use and illness chronicity. For analysis, per-round difference scores were computed as expected minus immediate for each valence and averaged within loss/win categories. Between-group comparisons used two-tailed independent-samples tests with α = .05 and Cohen’s d as the effect-size index.

**Results:**

We compared the expected–immediate difference scores between groups. For loss rounds, the difference in negative affect (expected minus immediate) was larger in the control group than in the SBD group (t = 2.96, p = .005, Cohen’s d = 0.80). For positive affect, the expected–immediate difference showed no between-group differences.

**Conclusions:**

Findings are consistent with blunted loss reactivity in SBD as a potential pathway influencing risky decision-making. Design features (round-level ratings, fixed outcomes) and sample size maybe limiting generalizability. Results should be replicated with larger cohorts to clarify when the expected–immediate gap impacts behavior and how it can inform interventions targeting emotional awareness and emotion regulation during decision-making.

**Disclosure of Interest:**

None Declared

